# Glyceryl Trinitrate for Prevention of Post-ERCP Pancreatitis and Improve the Rate of Cannulation: A Meta-Analysis of Prospective, Randomized, Controlled Trials

**DOI:** 10.1371/journal.pone.0075645

**Published:** 2013-10-01

**Authors:** Jiexia Ding, Xi Jin, Yue Pan, Shan Liu, Youming Li

**Affiliations:** Department of Gastroenterology, The First Affiliated Hospital, College of Medicine, Zhejiang University, Hangzhou, Zhejiang, China; The Chinese University of Hong Kong, Hong Kong

## Abstract

**Background:**

Acute pancreatitis is the most common complication of diagnostic and therapeutic endoscopic retrograde cholangiopancreatography (ERCP). Several clinical trials used glyceryl trinitrate (GTN) to prevent the incidence of post-ERCP pancreatitis (PEP). However, the results were still controversial.

**Objective:**

To conduct a meta-analysis of published, full-length, randomized controlled trials evaluating the effect of prophylactic GTN on the prevention of PEP, improve the rate of cannulation and the prevention of hyperamylasemia.

**Methods:**

Literature searches were conducted using PubMed, EMBASE, The Cochrane Library and Web of Knowledge databases, using keywords "post-ERCP" and "pancreatitis" and limited in randomized controlled trials.

**Results:**

Twelve RCTs involving 2649 patients were included. Eleven RCTs compared GTN with placebo for PEP prevention. Meta-analysis showed the overall incidence of PEP was significantly reduced by GTN treatment (RR 0.67; 95% CI, 0.52-0.87). Nevertheless, GTN administration did not decrease the incidence of moderate to severe PEP (RR 0.70; 95% CI, 0.42-1.15). Subgroup analyses revealed that GTN administered by sublingual was more effective than transdermal and topical in reducing the incidence of PEP. Besides, the prophylactic effect of GTN was far more obvious in the group of high PEP incidence than in the group of low PEP incidence. Additionally, the incidence of hyperamylasemia was significantly reduced by GTN treatment (RR 0.69; 95% CI, 0.54-0.90). No differences of the successful cannulation rate of bile ducts (RR 1.03; 95% CI, 0.99-1.06) attributable to GTN were observed.

**Conclusion:**

Prophylactic use of GTN reduced the overall incidence of PEP and hyperamylasemia. However, GTN was not helpful for the severity of PEP and the rate of cannulation.

## Introduction

Pancreatitis remains the most common severe complication of endoscopic retrograde cholangiopancreatography (ERCP) [1]. The incidence of post-ERCP pancreatitis (PEP) had been growing quickly for 30 years, varying from <2% up to 40% [2–4]. Although most PEP was mild, severe pancreatitis also occurred. Despite attempting to address this problem, effective strategies to prevent this serious complication remained elusive. Accumulating data revealed that risk factors associated with PEP development include both patient-related factors (female, sphincter of Oddi dysfunction(SOD), previous pancreatitis, chronic pancreatitis absent, age <60 years old and normal bilirubin) and procedure-related factors (precut sphincterotomy, pancreatic duct injection, balloon dilation of intact sphincter, pancreatic sphincterotomy, difficult cannulation, minor papilla sphincterotomy, pain during ERCP and ampullectomy) [5]. Currently, the pathogenesis of ERCP-induced pancreatitis has not been completely clarified. During diagnostic and therapeutic ERCP, the pancreas is exposed to multiple potentially damaging factors, including mechanical, hydrostatic, chemical, enzymatic, and microbiological etiologies. The exact mechanisms by which these factors trigger pancreatitis are unknown [6].

The ideal pharmacological drug should be highly effective in reducing PEP, have a short administration time, well tolerated with a low side-effect profile and cost-effective. Numerous pharmacological drugs of preventing PEP have been investigated, including non steroidal anti-inflammatory drugs (NSAID), diclofenac, ceftazidime, octreotide, protease inhibitors and heparin and so on. However, most results were disappointing and currently no pharmacological prophylaxis for PEP is in routine use [5]. In the human gastrointestinal tract, non-adrenergic non-cholinergic (NANC) innervation is important in nerve mediated relaxation and membrane hyperpolarisation and accumulating evidences indicate that nitric oxide (NO) is a NANC neurotransmitter. NO is synthesised from L-arginine by the enzyme nitric oxide synthase (NOS). It then activates soluble guanylate cyclase and catalyses formation of cyclic GMP that is an inhibitor of smooth muscle contraction. GTN, an NO donor, interacts with intracellular sulfhydryl groups (-SH) and formation of NO, inhibits sphincter of Oddi (SO) tonic and phasic contraction. This mechanism may be accounted for the prevention of PEP [7]. Three meta-analyses advocated the efficacy of GTN in PEP prevention [8–10] while another meta-analysis showed opposite result [11]. Except for these published meta-analyses, three additional trials provided inconsistent data in this area [12–14]. Of note, all these three trials had negative findings. Therefore, whether GTN can be used for PEP prophylaxis is still controversial. To tackle this controversy, we conducted an updated and comprehensive meta-analysis with inclusion of the newly published randomized, controlled trials (RCTs) to examine the efficacy of prophylactically administered GTN on PEP prevention, successful cannulation rate of bile ducts and hyperamylasemia prevention.

## Methods

### Study identification and selection

Literature searches of the electronic databases included PubMed, EMBASE, ISI Web of Knowledge (up to May 2013) and the Cochrane Library (Issue 4 of 12, Apr 2013). The search terms included “endoscopy,” “ERCP,” “endoscopic retrograde cholangiopancreatography,” “post-ERCP pancreatitis,” “post-endoscopic retrograde cholangiopancreatography pancreatitis,” “pancreatitis,” “PEP,” “cannulation,” “GTN,” “glyceryl trinitrate,” “nitroglycerin,” “glyceryl nitrate,” and “randomized controlled trial”. No language restriction was imposed. The manual searching of reference lists from potentially relevant papers was performed to identify any additional studies that have been missed from using the computer-assisted strategy. The following selection criteria were applied: (1) study design: prospective, randomized, controlled trials; (2) study population: patients undergoing ERCP; (3) intervention: prophylactic administration of GTN; (4) comparison intervention: placebo or no treatment; and (5) outcome measures: the overall incidence of PEP, the incidence of moderate to severe PEP, the successful rate of cannulation and the incidence of hyperamylasemia.

### Study quality analysis and data extraction

Two independent reviewers (D.J.X. and J.X.) assessed the quality score of primary trials according to the Jadad scale [15]. The quality scale ranges from 0 to 5 points. Higher scores indicate better reporting. We defined studies with a Jadad score of 3 points and higher as high quality in this meta-analysis. Disagreements were discussed by the reviewers and resolved through consensus. Data from eligible studies were extracted independently by two reviewers (D.J.X. and J.X.) using standard forms. Details of the studies including first author, year of publication, country, setting, sample size, interventions, dosage, follow-up, routes of drug administration, inclusion and exclusion criteria of each study, definition, incidence of PEP (including overall and moderate to severe pancreatitis, respectively), incidence of cannulation and hyperamylasemia. Any disagreements were resolved by discussion and consensus.

### Statistical analysis

All statistical analyses were performed using Review Manager (Version 5.1, Cochrane Collaboration, Oxford, UK). All outcomes were expressed as risk ratio (RR) with 95% CI. Heterogeneity was assessed by visual inspection of a forest plot, the Cochran Q test, and the I^2^ statistic. Heterogeneity was considered significant by the Cochran Q test with P<0.05 or by I^2^ greater than 50% [16,17]. A fixed-effects model or random-effects model was used, depending on the absence or presence of heterogeneity. We performed a sensitivity analysis by removing one study in turn from the overall data to evaluate the influence of a single study on the pooled analysis and by restricting the meta-analysis to several subgroups: the route of GTN administration (topical vs. transdermal vs. intravenous vs. sublingual) and the incidence of PEP in control arms(high incidence vs. low incidence). We also assessed the potential for publication bias shown as a funnel plot. A P value less than 0.05 was judged as statistically significant.

## Results

### Study selection and characteristics

177 potential RCTs were identified through in-depth search. Among them, 41 RCTs were excluded because of duplicate studies and 119 RCTs were excluded based on the titles and abstracts (meta-analysis or not relevant to our analysis). The remaining 17 were then retrieved for full text review. Finally, twelve fully published RCTs met inclusion criteria and were included in this meta-analysis [12–14,18–26]. Two studies were multicentre trials involving 14 [25] and 20 [24] centers. The principal characteristics of the included RCTs were shown in Table 1 and Table 2. Of the twelve studies, three were conducted in Asia, one in Oceanica and eight in Europe. The routes of GTN administration were transdermal, sublingual, intravenous and topical. All patients received GTN before ERCP. Nine trials [12–14,18,19,21,23–25] defined PEP with consensus criteria. One trial defined PEP as high-amylase value over the normal value after ERCP, did not indicate how much times than the upper limit normal [26]. One trial only analyzed the rate of cannulation, without showing the definition of PEP [20]. One trial did not show the definition of PEP but analyzing PEP [22]. Seven trials [14,19,21,23–26] used the Cotton criteria to assess pancreatitis severity, while the remaining three trials did not specify the definition of pancreatitis severity [12,13,22]. Seven and three RCTs were included for meta-analysis of the effect of GTN on the cannulation of bile ducts [12,13,18–24] and the hyperamylasemia [12,14,26], respectively.

**Table 1:** Principal characteristics of the published randomized studies included in the meta-analysis.

Group (year of publication)	Recruiting centres(n)	Location	Number of patients (Treatment/Control)	Mean age (Treatment/Control)	Male (%)	Route	Intervention (Treatment/Control)	Follow-up	PEP in GTN group, %(number)	PEP in control group, %(number)	Cannulation in treatment group, %(number)	Cannulation in control group, %(number)
Sudhindran (2001)[18]	1	UK	186 (90/96)	63.7 (63.7/63.7)	31 (24/37.5)	sublingual	GTN 2 mg, 5 min before ERCP/placebo	24h	7.8 (7/90)	17.7 (17/96)	93 (84/90)	92 (88/96)
Wehrmann (2001)[19]	1	Germany	80 (40/40)	58.72 (58.93/58.5)	45 (40/50)	topical	GTN 10 mg at ERCP/ physiological saline	24h	Mild:7.5 (3/40)	Mild:10 (4/40)	75 (30/40)	72.5 (29/40)
Ghori A (2002)[20]	1	UK	254 (128/126)	66 (67/65)	36 (35/37)	sublingual	GTN 0.4-0.8 mg/placebo	Not clear			93 (119/128)	84.2 (106/126)
Moretó M (2003)[21]	1	Spain	144 (71/73)	66 (66.7/65.2)	60 (62/59)	transdermal	GTN 15 mg, 30–40min befor ERCP/placebo	24h	4.3 (3/70) Moderate:1.4 (1/70)	15.3 (11/72) Moderate:1.39 (1/72)	94 (67/71)	93 (68/73)
Talwar A (2005)[22]	1	UK	104 (52/52)	64 (66/62)	31 (29/33)	topical	GTN 5 mg, before ERCP/physiological saline	Not clear	1.9 (1/52)	0(0/52)	90.4 (47/52)	86.5 (45/52)
Kaffes AJ (2006)[23]	1	Australia	318 (155/163)	62 (60/65)	39 (38/35)	transdermal	GTN 5 mg 60 minutes before ERCP/placebo	30 days	Totol:7.1(11/155) Mild:5.8 (9/155) Moderate:1.3 (2/155)	Totol:6.1(10/163) Mild:3.7 (6/163) Moderate:2.4 (4/163)	87 (135/155)	87 (142/163)
Beauchant M (2008)[24]	20	France	208 (105/103)	52 (50/54)	28 (28/28)	intravenous	GTN, Bolus of 0.1 mg at 10 min before ERCP, then 35 ug/kg/min for 6 h /placebo	1 month	Totol:9.5(10/105) Mild:2.9 (3/105) Moderate:4.7 (5/105) Sereve:1.9(2/105)	Totol:14.6(15/103) Mild:4.9 (5/103) Moderate:5.8 (6/103) Sereve:3.9(4/103)	92.4 (97/105)	94.2 (97/103)
Nøjgaard C (2009)[25]	14	Norway, Denmark, Sweden, France	806 (401/405)	66 (67/65)	41 (41/41)	transdermal	GTN 15 mg/24h 30 to 45 minutes before ERCP/plcabo	14 days	4.5 (18/401) Mild:1 (4/401) Moderate:2.2 (9/401) Sereve:1.3(5/401) Died:1/401	7.2 (29/405) Mild:2.2 (9/405) Moderate:4.2 (17/405) Sereve:0.8(3/405) Died:1/405		
Hao JY (2009)[26]	1	China	74 (38/36)	63.8 (64.3/63.4)	42 (39/44)	sublingual	GTN 5 mg 5 min before ERCP/0.1g Vit C	24h	7.9 (3/38)	25 (9/36)		
Nashaat E (2010) [12]	1	Egypt	80 (40/40)		50 (50/50)	transdermal	GTN 15 mg 2 houur before ERCP/no intervention	72h	17.5 (7/40)	10 (4/40)	90 (36/40)	85 (34/40)
Bhatia V (2011)[13]	1	India	250 (124/126)	42 (42/42.5)	33 (29/37)	transdermal	GTN 10 mg/h 30 minutes before ERCP/no intervention	24h	Mild:9.7 (12/124)	Mild:10.3 (13/126)	87.9 (109/124)	86.5 (109/126)
Chen XW (2012)[14]	1	China	147 (74/73)	65 (66/64.1)	48 (51/45)	sublingual	GTN 0.5mg 5 min before ERCP, then 35 ug/kg/min for 6 h /0.1g Vit C	24h	Totol:12.2(9/74) Mild:10.8 (8/74) Moderate:1.4 (1/74)	Totol:20.5(15/73) Mild:19.2 (14/73) Moderate:1.3 (1/73)		

ERCP, endoscopic retrograde cholangiopancreatography; PEP, post-ERCP pancreatitis; GTN, Glyceryl trinitrate.

**Table 2:** Principal characteristics of the published randomized studies included in the meta-analysis.

Group	Inclusion criteria	Exclusion criteria	Definition of PEP
Sudhindran[18]	Age >18 years, undergo ERCP	Acute or chronic pancreatitis,use of nitrate-containing medication.	Abdominal or back pain and serum amylase >1000 (normal range 5-300) units⁄ ml 6 and 24 h after ERCP
Wehrmann[19]	Undergo ERCP, papilla was normal	Previous gastroduodenal or bilio-pancreatic surgery or a previous bile-duct cannulation attempt within 3 months before entry into the study. Use of any medication probably affecting SO motility.	Abdominal pain persisting for 24 h associated with a 3-fold increase in serum amylase and⁄ or lipase
Ghori A[20]	Undergo ERCP	Undergo previous sphincterotomy, sent insertion or gastric surgery.	Not clear
Moretó M[21]	Age >18 years, undergo ERCP	Hypersensitivity to nitrates, active acute pancreatitis, anemia, glaucoma, severe hypoxemia with unbalanced ventilation/perfusion , hypotension,Previous sphinterotomy, known tumor of the major duodenal papilla, Use of nitrates, etc.	Abdominal pain persisting for 24 h associated with a 3-fold increase in serum amylase and⁄ or lipase
Talwar A[22]	Age >18 years, undergo ERCP	Previous ERCP resulting in endoscopic sphincterotomy, needle-knife papillotomy, or stenting, Oral or sublingual nitrate use for angina, Patient refusal	Not clear
Kaffes AJ[23]	Age >18 years , with an intact papilla, undergo ERCP	Current nitrate users, hypotensive systolic blood pressure [SBP]<90 mm Hg, hypoxic oxygen saturation [SO2]<95 mm Hg on supplemental oxygen,hemodynamic instability inability to consent, prior adverse effects with nitrate compounds and sildenafil users	Abdominal pain and a greater than 3-fold elevation of serum amylase above the upper limit of normal at 24 hours after the procedure
Beauchant M[24]	Aged between 18 and 75years, undergo ERCP	Acute pancreatitis in the month before inclusion, or chronic pancreatitis or anampullary carcinoma, or if they needed pancreatic sphincterotomy and/or pancreatic stenting, or if their hemodynamic status was unstable, etc.	Epigastric pain and a rise in serum amylase and/or lipase concentration to more than three times the normal upper limit 24 hours after endoscopy
Nøjgaard C[25]	Age >18 years, ERCP procedure planned at the center, patient able to give informed consent	Acute or chronic pancreatitis, sphincterotomy, Hypotension, Anemia, Hypersensibility to GN, Sildenafil administration in the 24 hours before the ERCP procedure, etc	Pain and 3-fold elevated serum amylase
Hao JY[26]	Age >18 years, undergo ERCP	Acute or active chronic pancreatitis, a nitrate allergic history, and those undergone sphincterotomy	Abdominal pain and high-amylase value over the normal value after ERCP. Hyperlipidemia was defined as the higher serum amylase concentration without or only with mild abdominal pain.
Nashaat E[12]	undergo ERCP	Hypersensitivity to used drugs, Active acute pancreatitis, Hypotension, Patients with renal impairment, have peptic ulcer, Patients with previous sphincterotomy, ampullary or pancreatic cancer invading the papilla, ect	Pain and 3-fold elevated serum amylase
Bhatia V[13]	Age>18 years and under a first ERCP	Acute or chronic pancreatitis, lower end malignant bile duct block, ongoing therapy with nitrates, calcium channel blockers, angiotensin-converting enzyme inhibitors, β-blockers, diuretics, or tricyclic antidepressants, patients with angina pectoris , history of myocardial infarction, or cerebral ischemia; and history of allergy to sulfa drugs.	Presence of pain persisting for 24 hours post-ERCP, and associated with a rise in serum amylase levels to more than 3 times the upper limit of normal
Chen XW[14]	Age >18 years, undergo ERCP	Acute or chronic pancreatitis, Hypersensitivity to GTN, sphincterotomy, severe cardiovascular and cerebrovascular diseases, anemia	Abdominal pain persisting for 24 h associated with a 3-fold increase in serum amylase

SO, sphincter of Oddi.

### Quality assessment

All trials met at least three criteria for trial quality excepted one trial [23] (Table 3). Successful randomization were completed in all trials and ten trials assessment were double-blinded [13,18–26]. Withdrawals and dropouts were clearly reported in all trials. Eleven trials had equal use of co-intervention for treatment of two groups [12–14,18–22,24–26]. Follow-up was not complete in five reported trials [13,14,18,23,24] and six trials were not included in the final analysis on an intention-to-treat basis due to loss of follow-up or excluded from analysis of PEP [13,14,18,21,23,24]. All trials had high quality in meta-analysis, where seven trials got 5 points [13,14,18,19,22,23,25], four trials got 4 points [20,21,24,26] and one trial had 3 points [12].

**Table 3:** Jadad quality scores of randomized controlled trials included in meta-analysis.

Group	Randomization	Blind	withdrawals and dropouts	Jadad score
Sudhindran[18]	computer generated number	double	clear reported	5
Wehrmann[19]	computer generated number	double	clear reported	5
Ghori A[20]	not clear randomized	double	clear reported	4
Moretó M[21]	not clear randomized	double	clear reported	4
Talwar A[22]	computer generated number	double	clear reported	5
Kaffes AJ[23]	computer generated randomization protocol	double	clear reported	5
Beauchant [24]	not clear randomized	double	clear reported	4
Nøjgaard C[25]	computer-generated randomization code	double	clear reported	5
Hao JY[26]	not clear randomized	double	clear reported	4
Nashaat E[12]	not clear randomized	not clear	clear reported	3
Bhatia V[13]	computer-generated random numbers	double	clear reported	5
Chen XW[14]	randomize table generated random numbers	single	clear reported	5

### PEP incidence analysis

**Figure 1:**
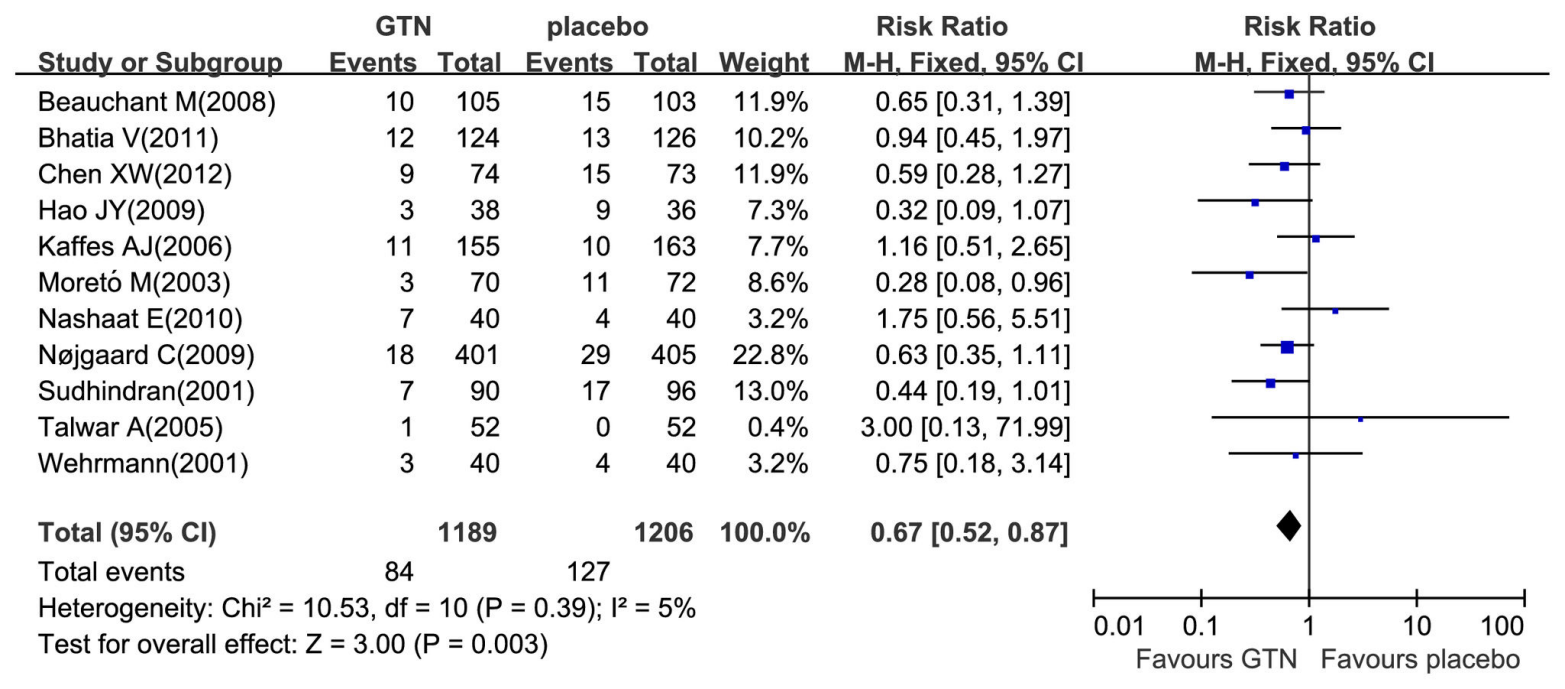
Meta-analyses between GTN and PEP. Forest plot demonstrated a significant decrease in the overall incidence of PEP with prophylactic GTN use. CI, confidence interval; M-H, Mantel-Haenszel; GTN, glyceryl trinitrate.

**Figure 2:**
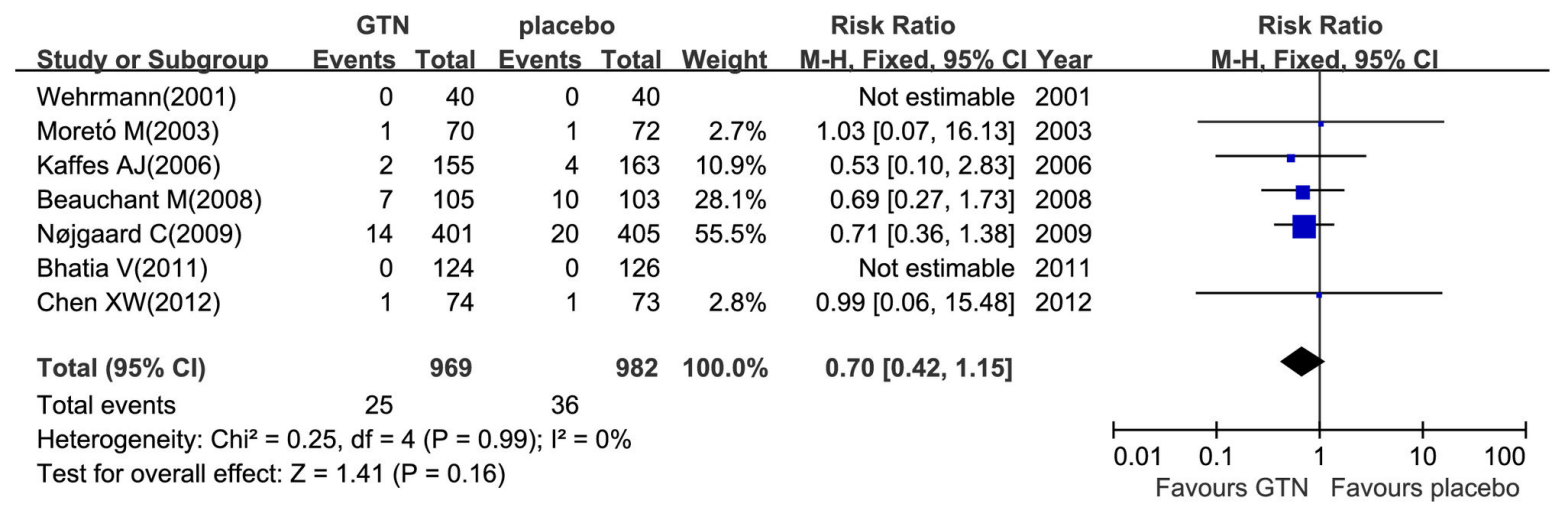
Meta-analyses between GTN and PEP. Forest plot demonstrated no significant decrease in the incidence of moderate to severe PEP with prophylactic GTN use. CI, confidence interval; M-H, Mantel-Haenszel; GTN, glyceryl trinitrate.

A total of eleven studies reported PEP [12–14,18,19,21–26] and 8.8% (211/2395) patients developed PEP, where 7.1% (84/1189) in the GTN group and 10.5% (127/1206) in the placebo group. There was no heterogeneity among these studies (P_heterogeneity_ =0.39, I^2^= 5%). So we used the fixed-effects model and found that the administration of GTN was associated with a significant reduction in the overall PEP incidence (RR 0.67; 95% CI, 0.52-0.87; P =0.003) (Figure 1), showing that PEP incidence was significantly lower in the treatment group than in the placebo group. Seven studies reported moderate to severe PEP [12,14,19,21,23–25] and 3.1% (61/1951) patients developed moderate to severe PEP, where 2.6% (25/969) in the GTN group and 3.7% (36/982) in the placebo group, respectively. Heterogeneity was not evident among these seven studies (P_heterogeneity_ =0.99, I^2^=0%). Therefore, we pooled the results by the fixed-effects model. Overall result showed no significant reduction in the incidence of moderate to severe PEP (RR 0.70; 95% CI, 0.42-1.15; P=0.16) (Figure 2).

### Subgroup and sensitivity analysis

Different routes of GTN administration may influence its effectiveness. Based on this hypothesis, we performed subgroup meta-analysis of these trials (Table 4). The subgroup analyses suggested that topical and transdermal application may not be useful for PEP reduction (RR 1.00; 95% CI,0.28-3.53; P=1.00; I^2^=0%, P_heterogeneity_ =0.43 and RR 0.78; 95% CI, 0.55-1.10; P=0.16; I^2^=36%, P_heterogeneity_ =0.18, respectively), whereas the sublingual route was associated with statistically significantly reduced rates of PEP (RR 0.47; 95% CI, 0.28-0.78;P = 0.003; I^2^=0%, P_heterogeneity_ =0.68) (Figure S1). Because there was only one trial that used the intravenous route [24], no subgroup analysis was conducted within this group of patients. In this study [24], GTN offered a limited and clinically nonsignificant benefit for PEP prevention. Through analyzing different route of administration of GTN, we concluded that sublingual GTN administration is more effective than transdermal and topical GTN administration for PEP prevention. There were no heterogeneities of these three subgroup analysis, so we used fixed-effect model. We suggested that investigation regarding the sublingual form for preventing PEP should be paid more attention to, and more RCTs should be performed to further confirm the effect of sublingual form on PEP.

**Table 4:** Subgroup and sensitivity analysis of the prophylactic effect of GTN on the incidence of PEP.

Subgroup	Patients	RR (95%CI)	Z	P	Heterogeneity		
					x^2^	I^2^	P
Topical route	184	1.00 [0.28, 3.53]	0.00	1.00	0.61	0%	0.43
Sublingual route	407	0.47 [0.28, 0.78]	2.93	0.003	0.79	0%	0.68
Transdermal route	1596	0.78 [0.55, 1.10]	1.42	0.16	6.22	36%	0.18
Low incidences of PEP	1638	0.88 [0.62, 1.26]	0.69	0.49	3.80	0%	0.58
High incidences of PEP	757	0.48 [0.32, 0.71]	3.63	0.00003	2.16	0%	0.71
Excluded one study[22]	2291	0.66 [0.51, 0.86]	3.08	0.002	9.69	7%	0.38
Excluded two studies[22,26]	2217	0.69 [0.53, 0.90]	2.71	0.007	8.15	2%	0.42

The reviewers further decided to perform a subgroup analysis of the effect of GTN on patients stratified according to the incidence of PEP in the control groups (Figure S2). Because the overall incidence of PEP in the control group was 10.5%, we finally took 10.5% as the cutoff point to stratify the trials. The subgroup analysis (Table 4) revealed that GTN may not be useful for PEP reduction in trials with a low PEP incidence in the control group (RR 0.88; 95% CI, 0.62-1.26; P=0.49; I^2^=0%; P_heterogeneity_=0.58). However, in trials with a high pancreatitis incidence in control group, there was a significant reduction of PEP in the GTN group (RR 0.48; 95% CI, 0.32-0.71; P=0.0003; I^2^=0%; P_heterogeneity_=0.71). The sensitive analysis by excluding study of unclear PEP definition [22] also yielded a significant result (RR 0.66; 95% CI, 0.51-0.8; P=0.002) and had no heterogeneity (P_heterogeneity_ =0.38, I^2^= 7%). We further excluded another trial [26], where PEP definition was not in accordance with other trials. After the studies excluded, we also yielded a significant result (RR 0.69; 95% CI, 0.53-0.90; P=0.007) and had no heterogeneity (p_heterogeneity_ =0.42, I^2^= 2%) (Table 4).

### The effect of GTN on the successful rate of cannulation of bile ducts and hyperamylasemia prevention

**Figure 3:**
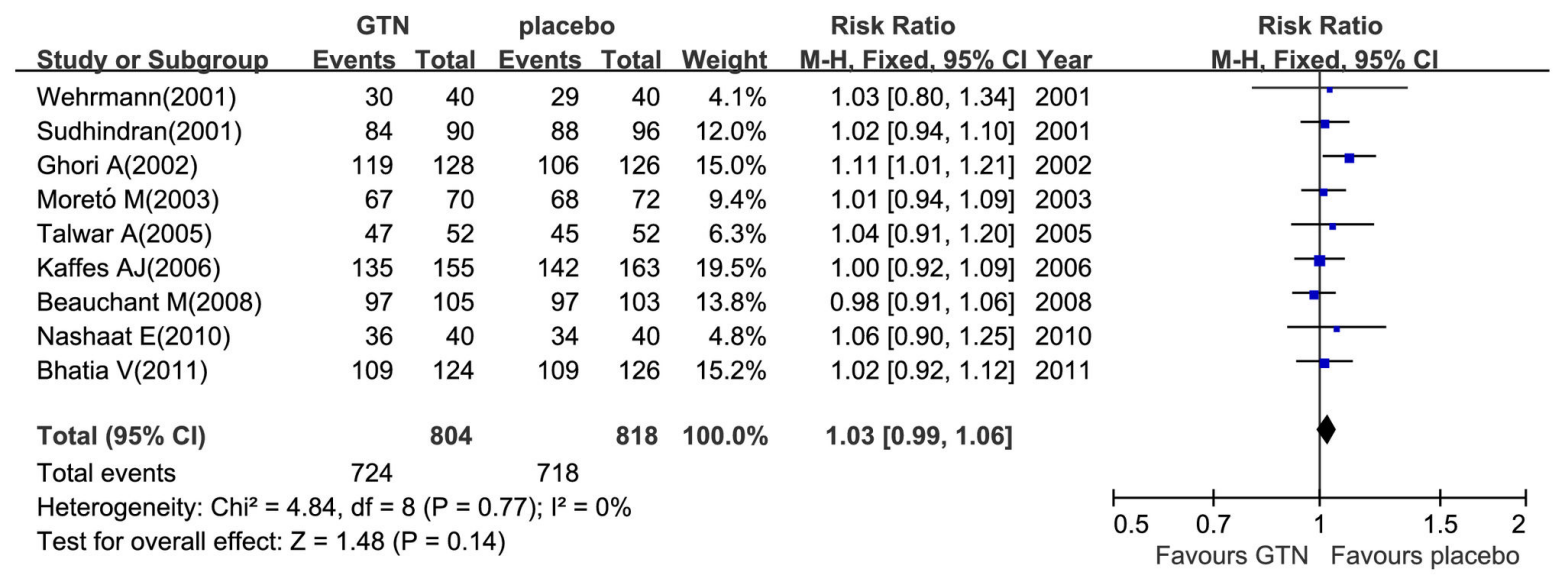
Meta-analyses between GTN and cannulation. Forest plot showed no helpful for increasing the successful rate of cannulation of bile ducts with prophylactic GTN use. CI, confidence interval; M-H, Mantel-Haenszel; GTN, glyceryl trinitrate.

**Figure 4:**
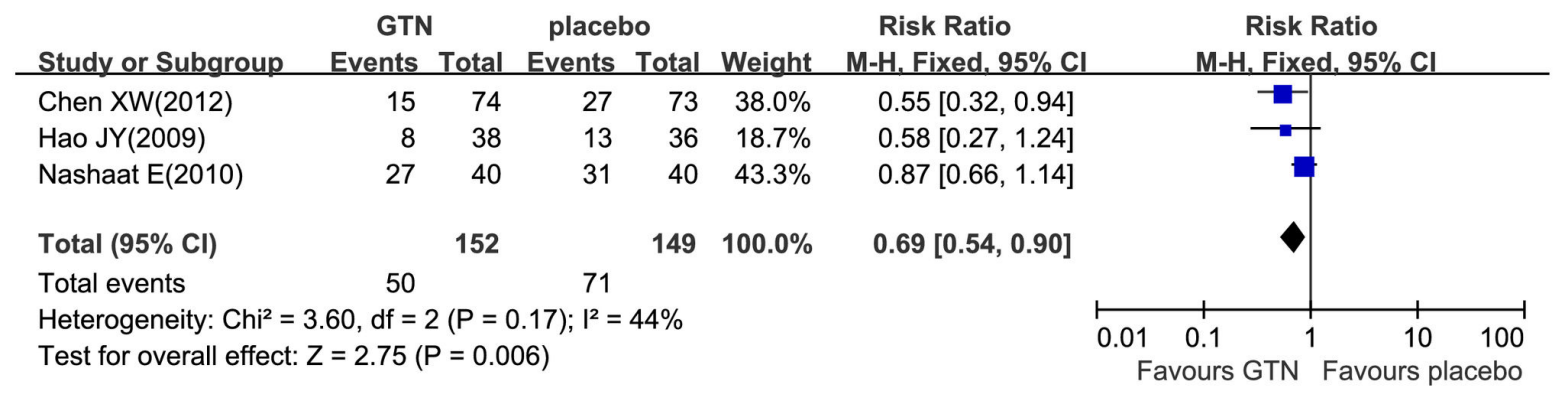
Meta-analyses between GTN and hyperamylasemia. Forest plot demonstrated a significant decrease in the incidence of hyperamylasemia with prophylactic GTN use. CI, confidence interval; M-H, Mantel-Haenszel; GTN, glyceryl trinitrate.

A total of 1622 patients were included in the nine trials comparing GTN with placebo in the successful rate of cannulation [12,13,18–24], and there were no homogeneity with included trials (I^2^=0%, P_heterogeneity_ =0.77). Altogether, 88.9% (1442/1622) patients had successful cannulation, of which 90% (724/804) was in the GTN group and 87.8% (718/818) in the placebo group (Figure 3). The meta-analysis indicated no significant benefit of the successful rate of cannulation with GTN use (RR 1.03; 95% CI, 0.99–1.06; P=0.14). Furthermore, only 301 patients included in the three trials comparing GTN with placebo in the incidence of hyperamylasemia [12,14,26]. There were no homogeneous with included trials (I^2^=44%, P_heterogeneity_=0.17). Altogether, 40.2% (121/301) of patients had hyperamylasemia, of which 32.9% (50/152) was in the GTN group and 47.7% (71/149) in the placebo group (Figure 4). The meta-analysis indicated a significant reduced incidence of hyperamylasemia with GTN use (RR 0.69; 95% CI, 0.54–0.90; P = 0.006).

### Adverse effect

Eight trials reported the adverse events that were potentially related to GTN [13,14,18,20,21,23–25]. Six studies [13,14,18,21,24,25] reported that 12.5% (217/1739) patients had hypotension (Figure S3), of which 20.5% (177/864) was in the GTN group and 4.6% (40/875) in the control group. As it had heterogeneity (P_heterogeneity_= 0.0001; I^2^ = 80%), we changed to random-effect model. GTN use significantly increased the risk of hypotension (RR 5.88; 95% CI, 1.88-18.39; P=0.002). In addition, six studies [13,14,21,23–25] reported that 8.8% (164/1871) patients had headache (Figure S4), where 13.7% (127/929) in the GTN group and 3.9% (37/942) in the control group. For there was moderate heterogeneity among these trials (P_heterogeneity_ = 0.07; I^2^ = 51%), we used fixed-effect model. GTN use significantly increase the risk of headache (RR 3.45; 95% CI, 2.45-4.86; P<0.00001). Different routes of GTN administration may have different risk of adverse effect. Based on this hypothesis, we performed subgroup analysis of these trials (Table S1). The subgroup analyses suggested that compared to sublingual and transdermal GTN administration, the intravenous GTN administration had the highest risk of hypotesion (64.8% vs. 54.9% vs. 3.2%) and headache (33.3% vs. 4.1% vs. 11.9%). Two studies [13,21] reported seven cases of vomiting or nausea, where 3.1% (6/195) was in the GTN group and 0.5% (1/199) in the control group. Kaffes AJ,et al [23] showed rash in both groups, but had no difference. Four trials [12,19,22,26]did not report adverse events, where two [19,22]of them were in the topical route of GTN.

### Publication bias

**Figure 5:**
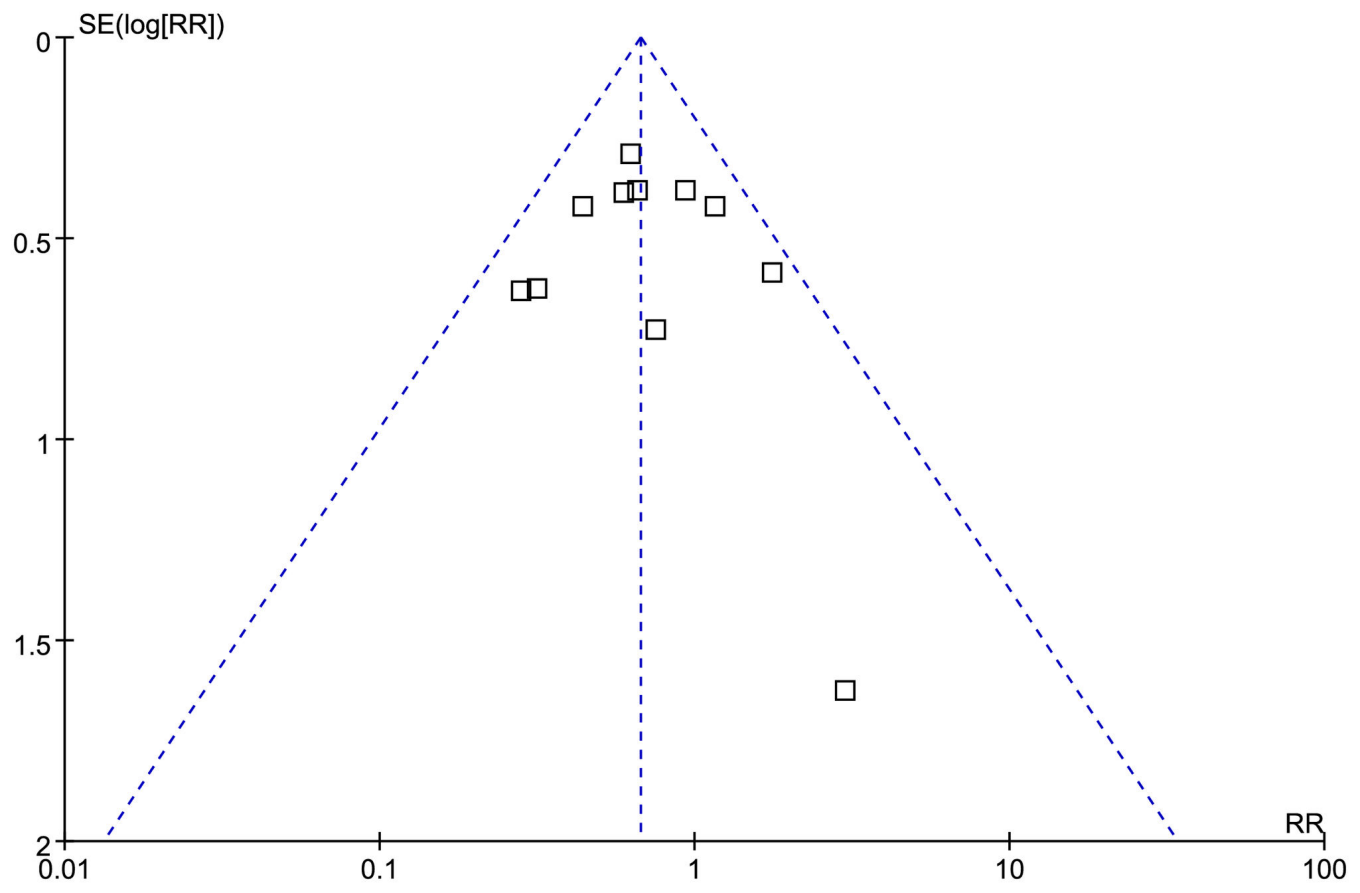
Funnel plot for publication bias in the risk ratio (RR) analysis. Each dot represented the RRs for the percentage of the incidence of PEP with prophylactic GTN use or placebo use. The dashed line represents the 95% CI line.

The funnel plot showed that there was no potential publication bias among these included trials (Figure 5). The dots distributed on both sides of a dashed line, showing that the negative or null studies were located.

## Discussion

Meta-analysis of the eleven included RCTs demonstrates that the RR of PEP developing after prophylactic GTN use was 0.67(95% CI, 0.52-0.87). In other words, patients who received GTN in the periprocedural period were 33% less likely to have pancreatitis. However, GTN-treated patients did not have a reduction of the development of moderate to severe PEP. Though our findings were similar to three previous meta-analyses published on this topic [8–10], our meta-analysis was markedly larger than previous analyses and up to date. Totally, there were three positive outcome trials [18,21,26], six negative outcome trials [12–14,23–25] and other two trials [19,22] without significant differences between the two arms. After adding three negative outcome trials in this meta-analysis, the PEP prevention effect of GTN was still concluded from this meta-analysis. In contrast to our study, one previous meta-analysis showed no significant reduction the incidence of PEP with GTN use [11]. This was probably attributable to the small number of only four RCTs included in that meta-analysis. More importantly, we found that the protective role of GTN was more obvious in centers with high PEP incidence than in centers with low PEP incidence, implying that GTN may have their greatest benefit for those high-risk patients or procedures and for those centers without experienced endoscopists and/or advanced instruments.

Four routes of GTN used in the trials: topical, sublingual, transdermal and intravenous. Whether the route of GTN administration affects the clinical efficacy was uncertain. From clinical point of view, three studies [14,18,26]assessing sublingual administered GTN to prevent PEP had positive results or demonstrated a trend toward positivity. In contrast, among five studies [12,13,21,23,25]assessing transdermal GTN adminstration, only one trial had positive results and other four trails had negative results or demonstrated a trend toward negative. Besides, two trials [19,22]and only one trial [24] respectively assessing topical and intravenous GTN adminstration, but all had negative results or demonstrated a trend toward negative. By summarizing the available evidence, the sublingual route of GTN administration seemed to be the best way for PEP prevention. Though the sublingual route seemed to be more effective than the transdermal route, the results were not convincing for limited patients in sublingual route (407 patients) compared with transdermal route (1596 patients).

The present meta-analysis suggested a significant benefit of GTN in PEP prevention (P = 0.003). The subsequent sensitive analysis by exclusion of two studies [22,26] in which the definition of PEP was not consistent with other trials, also yielded a significant result (P = 0.002, P=0.007, respectively). Sensitivity analysis suggested that there were no significant heterogeneity among the studies. Three trials had unusually high rates of PEP in the placebo groups in patients (17.7% [18] 25% [26] and 20.5% [14], respectively) and the subgroup analysis of trials with high risk of PEP suggested a statistical significance.

There were several hypothesis of ERCP-induced pancreatitis but is not completely understood [27,28]. The papillary instrumentation during ERCP may cause a spasm of the SOD and result in transient pancreatic duct obstruction and subsequent development of PEP [29]. It was demonstrated that GTN, a nitric oxide donor, lowered basal pressure and contraction amplitude in the SO [30]. Luman W, et al, reported local application of GTN inhibited SO motility and concluded that this may imply the application of GTN for diagnostic and therapeutic biliary endoscopy [7]. However, our analysis suggested that prophylactic use of GTN before ERCP seemed useless in increasing the successful cannulation rate of bile ducts. Three trials [12,14,26]reported the incidence of hyperamylasemia with prophylactic of GTN administration through transdermal and sublingual routes. Prophylactic GTN administration significantly reduced the incidence of hyperamylasemia.

NO is a reactive nitrogen species (RNS) while GTN is an NO donor. In pancreas cells, many RNS might reactive oxygen to produce peroxy nitrate and damage the cells. Establishment of redox balance is highly complicated, requiring sophisticated regulation of scavenger bioavailability and of RNS generation. The major cellular RNS scavenger in the pancreas is GSH (a tripeptide consisting of glutamate, cysteine, and glycine). The thiol group in the cysteine moiety of GSH accounts for its scavenging power. GSH concentration in the pancreas ranks the fourth highest among the visceral organs. Pancreatic GSH turnover is less only than that in the kidney and liver, which have twofold and fourfold the turnover rates of the pancreas, respectively. Therefore, it appears that the pancreas is “evolutionally prepared” for defense against oxidative stress and removal of RNS. Although GSH is the major cellular antioxidant in the pancreas, other cellular antioxidants are also present in the pancreas. In particular, vitamin C, vitamin E, and vitamin A are present in the pancreas in considerable amounts. These antioxidants may also be responsible for cellular defense against oxidative stress. Therefore, pancreas cells would not be damaged by GTN due to redox balance [31].

There were two ways commonly used to prevent PEP: pharmacological and procedural interventions. ERCP should be avoided in unnecessary or low yield cases, especially when multiple patient-related risk factors for pancreatitis development were found. A number of pharmacological drugs, in particular rectal NSAIDs [32], have also shown prospects but none are currently being consistently used. The procedural interventions that have been demonstrated to reduce PEP incidence include guide-wire cannulation [33] and pancreatic stent placement [34] in high-risk cases. However, surveys of physician practices showed that more than 20% of endoscopists did not perform prophylactic pancreatic stenting in any situations, which was mainly due to the lack of experience [35]. However, this method requires an experienced endoscopist, as failed pancreatic stenting carries a high risk of severe pancreatitis [36].

The ideal pharmacological drugs should be highly effective in reducing post-ERCP pancreatitis, have a short administration time, well tolerated with a low side-effect profile and cost-effective. Several drugs have shown prospect. However, the vast majority have fallen short of these goals. Therefore, adverse effects of GTN should be weighed against its potential clinical benefit. The main adverse effects were transient hypotension and headache, more frequent in intravenous administration delivery of GTN, but did not severe and responded to conventional treatment. The side effects were significantly more frequent in the GTN group and led to dose reduction or cessation of infusion [24]. Therefore, compared with intravenous administration, sublingual or transdermal delivery of GTN may be much safer, well tolerated, and easier to administer. Though realizing GTN could prevent PEP incidence, we should pay attention to its adverse events. More importantly, the present meta-analysis did not have any publication bias and retrieved complete identified research.

By summarizing all the relevant RCTs published to date, the present meta-analysis confirmed the efficacy and relative safety of GTN. Patients who underwent ERCP, the sublingual route of administration GTN is recommended and should pay attention to its adverse events.

## Conclusions

In conclusion, this meta-analysis show that the prophylactic use of GTN have an effective and relative safe intervention for preventing PEP and hyperamylasemia, but show no effect of the severity of PEP and the successful rate of cannulation of bile ducts. Further well-designed placebo-controlled RCTs are needed to confirm the effect of GTN in preventing PEP.

## Supporting Information

Checklist S1 **PRISMA 2009 Checklist for this study.**
(DOC)

Figure S1 **Meta-analyses between route of GTN and PEP.** Forest plot demonstrated a significant decrease in the incidence of PEP of the sublingual route, but had no significant decrease of the topical or transdermal routes with prophylactic GTN use. CI, confidence interval; M-H, Mantel-Haenszel; GTN, glyceryl trinitrate.
(EPS)

Figure S2 **Meta-analyses between GTN and incidence of PEP.** Forest plot demonstrated a significant decrease in the incidence of PEP in high incidence of PEP group, but no significant prevention of PEP in low incidence of PEP group with prophylactic GTN use. CI, confidence interval; M-H, Mantel-Haenszel; GTN, glyceryl trinitrate.
(EPS)

Figure S3 **Meta-analyses between GTN and hypotension.** Forest plot showed significantly increase the risk of hypotension with prophylactic GTN use. CI, confidence interval; M-H, Mantel-Haenszel; GTN, glyceryl trinitrate.
(EPS)

Figure S4 **Meta-analyses between GTN and headache.** Forest plot showed significantly increase the risk of headache with prophylactic GTN use. CI, confidence interval; M-H, Mantel-Haenszel; GTN, glyceryl trinitrate.
(EPS)

Table S1 **Analysis of the side effect profile by route of GTN administration.**
(DOC)

## References

[1] Arata S, Takada T, Hirata K, Yoshida M, Mayumi T et al. (2010) Post-ERCP pancreatitis. J Hepatobiliary Pancreat Sci 17: 70-78. doi:10.1007/s00534-009-0220-5. PubMed: 20012323. 20012323 10.1007/s00534-009-0220-5

[2] Testoni PA, Mariani A, Giussani A, Vailati C, Masci E et al. (2010) Risk factors for post-ERCP pancreatitis in high- and low-volume centers and among expert and non-expert operators: a prospective multicenter study. Am J Gastroenterol 105: 1753-1761. doi:10.1038/ajg.2010.136. PubMed: 20372116. 20372116 10.1038/ajg.2010.136

[3] Gottlieb K, Sherman S (1998) ERCP and biliary endoscopic sphincterotomy-induced pancreatitis. Gastrointest Endosc Clin N Am 8: 87-114. PubMed: 9405753. 9405753

[4] Freeman ML (2002) Adverse outcomes of ERCP. Gastrointest Endosc 56: S273-S282. doi:10.1016/S0016-5107(02)70025-4. PubMed: 12447281. 12447281 10.1067/mge.2002.129028

[5] Donnellan F, Byrne MF (2012) Prevention of Post-ERCP Pancreatitis. Gastroenterol. Res Pract: 796751. 10.1155/2012/796751 PMC3154520 21845187

[6] Abdel AA, Lehman GA (2007) Pancreatitis after endoscopic retrograde cholangio-pancreatography. World J Gastroenterol 13: 2655-2668. PubMed: 17569133. 17569133 10.3748/wjg.v13.i19.2655 PMC4147113

[7] Luman W, Pryde A, Heading RC, Palmer KR (1997) Topical glyceryl trinitrate relaxes the sphincter of Oddi. Gut 40: 541-543. PubMed: 9176086. 9176086 10.1136/gut.40.4.541 PMC1027132

[8] Bai Y, Xu C, Yang X, Gao J, Zou DW et al. (2009) Glyceryl trinitrate for prevention of pancreatitis after endoscopic retrograde cholangiopancreatography: A meta-analysis of randomized, double-blind, placebo-controlled trials. Endoscopy 41: 690-695. doi:10.1055/s-0029-1214951. PubMed: 19670137. 19670137 10.1055/s-0029-1214951

[9] Bang UC, Nøjgaard C, Andersen PK, Matzen P (2009) Meta-analysis: nitroglycerin for prevention of post-ERCP pancreatitis. Aliment Pharmacol Ther 29: 1078-1085. doi:10.1111/j.1365-2036.2009.03978.x. PubMed: 19236312. 19236312 10.1111/j.1365-2036.2009.03978.x

[10] Chen B, Fan T, Wang C (2010) A meta-analysis for the effect of prophylactic GTN on the incidence of post-ERCP pancreatitis and on the successful rate of cannulation of bile ducts. BMC Gastroenterol 10. 10.1186/1471-230X-10-85 PMC2921391 20673365

[11] Shao LM, Chen QY, Chen MY, Cai JT (2010) Nitroglycerin in the Prevention of Post-ERCP Pancreatitis: A Meta-Analysis. Dig Dis Sci 55: 1-7. doi:10.1007/s10620-008-0709-9. PubMed: 19160042. 19160042 10.1007/s10620-008-0709-9

[12] HANM Nashaat E (2010) The value of transdermal Glyceryl Trinitrate in the prevention of post-ERCP pancreatitis in comparison to Octreotide and Diclofenac injection. Nat Science 8: 27-35.

[13] Bhatia V, Ahuja V, Acharya SK, Garg PK (2011) Randomized Controlled Trial of Valdecoxib and Glyceryl Trinitrate for the Prevention of Post-ERCP Pancreatitis. J Clin Gastroenterol 45: 170-176. doi:10.1097/MCG.0b013e3181eb600e. PubMed: 20717044. 20717044 10.1097/MCG.0b013e3181eb600e

[14] Xiao-wei C, Wan-dong H, Xiao-li Wu Qing-Ke H, Qi-huai Z et al. (2012) Nitroglycerin for prevention of post-ERCP pancreatitis and hyperamylasemia. Chin J Digest Endosc 29: 181-184.

[15] Jadad AR, Moore RA, Carroll D, Jenkinson C, Reynolds DJ et al. (1996) Assessing the quality of reports of randomized clinical trials: is blinding necessary? Control Clin Trials 17: 1-12. doi:10.1016/S0197-2456(96)90740-0. PubMed: 8721797. 8721797 10.1016/0197-2456(95)00134-4

[16] Higgins JP, Thompson SG (2002) Quantifying heterogeneity in a meta-analysis. Stat Med 21: 1539-1558. doi:10.1002/sim.1186. PubMed: 12111919. 12111919 10.1002/sim.1186

[17] Higgins JP, Thompson SG, Deeks JJ, Altman DG (2003) Measuring inconsistency in meta-analyses. BMJ 327: 557-560. doi:10.1136/bmj.327.7414.557. PubMed: 12958120. 12958120 10.1136/bmj.327.7414.557 PMC192859

[18] Sudhindran S, Bromwich E, Edwards PR (2001) Prospective randomized double-blind placebo-controlled trial of glyceryl trinitrate in endoscopic retrograde cholangiopancreatography-induced pancreatitis. Br J Surg 88: 1178-1182. doi:10.1046/j.0007-1323.2001.01842.x. PubMed: 11531863. 11531863 10.1046/j.0007-1323.2001.01842.x

[19] Wehrmann T, Schmitt T, Stergiou N, Caspary WF, Seifert H (2001) Topical application of nitrates onto the papilla of Vater: manometric and clinical results. Endoscopy 33: 323-328. doi:10.1055/s-2001-13687. PubMed: 11315893. 11315893 10.1055/s-2001-13687

[20] Ghori A, Hallisey M, Nwokolo C, Loft D, Fraser I (2002) The secret (GTN) of successful ERCP cannulation: A prospective randomised controlled study. J R Coll Surg Edinb 47: 634-637. PubMed: 12363191. 12363191

[21] Moretó M, Zaballa M, Casado I, Merino O, Rueda M et al. (2003) Transdermal glyceryl trinitrate for prevention of post-ERCP pancreatitis: A randomized double-blind trial. Gastrointest Endosc 57: 1-7. doi:10.1067/mge.2003.28. PubMed: 12518122. 12518122 10.1067/mge.2003.29

[22] Talwar A, Dare C, Pain J (2005) Does topical GTN on the sphincter of Oddi facilitate ERCP? A double-blind randomized control trial. Surg Endosc 19: 902-904. doi:10.1007/s00464-004-9166-5. PubMed: 15868252. 15868252 10.1007/s00464-004-9166-5

[23] Kaffes AJ, Bourke MJ, Ding S, Alrubaie A, Kwan V et al. (2006) A prospective, randomized, placebo-controlled trial of transdermal glyceryl trinitrate in ERCP: effects on technical success and post-ERCP pancreatitis. Gastrointest Endosc 64: 351-357. doi:10.1016/j.gie.2005.11.060. PubMed: 16923481. 16923481 10.1016/j.gie.2005.11.060

[24] Beauchant M, Ingrand P, Favriel JM, Dupuychaffray JP, Capony P et al. (2008) Intravenous nitroglycerin for prevention of pancreatitis after therapeutic endoscopic retrograde cholangiography: A randomized, double-blind, placebo-controlled multicenter trial. Endoscopy 40: 631-636. doi:10.1055/s-2008-1077362. PubMed: 18680075. 18680075 10.1055/s-2008-1077362

[25] Nøjgaard C, Hornum M, Elkjaer M, Hjalmarsson C, Heyries L et al. (2009) Does glyceryl nitrate prevent post-ERCP pancreatitis? A prospective, randomized, double-blind, placebo-controlled multicenter trial. Gastrointest Endosc 69: e31-e37. doi:10.1016/j.gie.2008.11.042. PubMed: 19410035. 19410035 10.1016/j.gie.2008.11.042

[26] Hao JY, Wu DF, Wang YZ, Gao YX, Lang HP et al. (2009) Prophylactic effect of glyceryl trinitrate on post-endoscopic retrograde cholangiopancreatography pancreatitis: A randomized placebo-controlled trial. World J Gastroenterol 15: 366-368. doi:10.3748/wjg.15.366. PubMed: 19140238. 19140238 10.3748/wjg.15.366 PMC2653335

[27] Freeman ML (2007) Pancreatic stents for prevention of post-endoscopic retrograde cholangiopancreatography pancreatitis. Clin Gastroenterol Hepatol 5: 1354-1365. doi:10.1016/j.cgh.2007.09.007. PubMed: 17981248. 17981248 10.1016/j.cgh.2007.09.007

[28] Cooper ST, Slivka A (2007) Incidence, risk factors, and prevention of post-ERCP pancreatitis. Gastroenterol Clin North Am 36: 259-276. doi:10.1016/j.gtc.2007.03.006. PubMed: 17533078. 17533078 10.1016/j.gtc.2007.03.006

[29] Cotton PB, Lehman G, Vennes J, Geenen JE, Russell RC et al. (1991) Endoscopic sphincterotomy complications and their management: an attempt at consensus. Gastrointest Endosc 37: 383-393. doi:10.1016/S0016-5107(91)70740-2. PubMed: 2070995. 2070995 10.1016/s0016-5107(91)70740-2

[30] Staritz M, Poralla T, Ewe K, Meyer ZBK (1985) Effect of glyceryl trinitrate on the sphincter of Oddi motility and baseline pressure. Gut 26: 194-197. doi:10.1136/gut.26.2.194. PubMed: 3917965. 3917965 10.1136/gut.26.2.194 PMC1432421

[31] Leung PS, Chan YC (2009) Role of Oxidative Stress in Pancreatic Inflammation. Antioxid Redox Signal 11: 135-165. doi:10.1089/ars.2008.2109. PubMed: 18837654. 18837654 10.1089/ars.2008.2109

[32] Ding X, Chen M, Huang S, Zhang S, Zou X (2012) Nonsteroidal anti-inflammatory drugs for prevention of post-ERCP pancreatitis: a meta-analysis. Gastrointest Endosc 76: 1152-1159. doi:10.1016/j.gie.2012.08.021. PubMed: 23164513. 23164513 10.1016/j.gie.2012.08.021

[33] Cheung J, Tsoi KK, Quan WL, Lau JY, Sung JJ (2009) Guidewire versus conventional contrast cannulation of the common bile duct for the prevention of post-ERCP pancreatitis: a systematic review and meta-analysis. Gastrointest Endosc 70: 1211-1219. doi:10.1016/j.gie.2009.08.007. PubMed: 19962504. 19962504 10.1016/j.gie.2009.08.007

[34] Sofuni A, Maguchi H, Itoi T, Katanuma A, Hisai H et al. (2007) Prophylaxis of post-endoscopic retrograde cholangiopancreatography pancreatitis by an endoscopic pancreatic spontaneous dislodgement stent. Clin Gastroenterol Hepatol 5: 1339-1346. doi:10.1016/j.cgh.2007.07.008. PubMed: 17981247. 17981247 10.1016/j.cgh.2007.07.008

[35] Dumonceau JM, Rigaux J, Kahaleh M, Gomez CM, Vandermeeren A et al. (2010) Prophylaxis of post-ERCP pancreatitis: a practice survey. Gastrointest Endosc 71: 931-939. 20226455. 10.1016/j.gie.2009.10.055 20226455

[36] Freeman ML, Overby C, Qi D (2004) Pancreatic stent insertion: consequences of failure and results of a modified technique to maximize success. Gastrointest Endosc 59: 8-14. doi:10.1016/S0016-5107(04)00202-0. PubMed: 14722540. 14722540 10.1016/s0016-5107(03)02530-6

