# Supplementary material for: Glyceryl Trinitrate for Prevention of Post-ERCP Pancreatitis and Improve the Rate of Cannulation: A Meta-Analysis of Prospective, Randomized, Controlled Trials
Source: PLoS One. 2013 Oct 1;8(10):e75645. doi: 10.1371/journal.pone.0075645 (PMC3787965; doi:10.1371/journal.pone.0075645)
Supplement: Table S1 — Analysis of the side effect profile by route of GTN administration. (DOC) [file pone.0075645.s006.doc]

**Table S1 Analysis of the side effect profile by route of GTN administration**

| Adverse effect | Sublingual route,%(number) | | Transdermal route, %(number) | | Intravenous route, %(number) | |
| --- | --- | --- | --- | --- | --- | --- |
|  | Placebo group | GTN group | Placebo group | GTN group | Placebo group | GTN group |
| Hypotension | 3.0(5/169) | 54.9(90/164) | 0.7(4/603) | 3.2(19/595) | 30.1(31/103) | 64.8(68/105) |
| Headache | 0(0/73) | 4.1(3/74) | 4.2(32/766) | 11.9(89/750) | 4.9(5/103) | 33.3(35/105) |
